# A Genetically-Encoded YFP Sensor with Enhanced Chloride Sensitivity, Photostability and Reduced pH Interference Demonstrates Augmented Transmembrane Chloride Movement by Gerbil Prestin (SLC26a5)

**DOI:** 10.1371/journal.pone.0099095

**Published:** 2014-06-05

**Authors:** Sheng Zhong, Dhasakumar Navaratnam, Joseph Santos-Sacchi

**Affiliations:** 1 Dept. of Surgery (Otolaryngology), Yale University School of Medicine, New Haven, Connecticut, United States of America; 2 Dept. of Neurobiology, Yale University School of Medicine, New Haven, Connecticut, United States of America; 3 Dept. of Neurology, Yale University School of Medicine, New Haven, Connecticut, United States of America; 4 Dept. of Cellular and Molecular Physiology, Yale University School of Medicine, New Haven, Connecticut, United States of America; University of Iowa, United States of America

## Abstract

**Background:**

Chloride is the major anion in cells, with many diseases arising from disordered Cl^−^ regulation. For the non-invasive investigation of Cl^−^ flux, YFP-H148Q and its derivatives chameleon and Cl-Sensor previously were introduced as genetically encoded chloride indicators. Neither the Cl^−^ sensitivity nor the pH-susceptibility of these modifications to YFP is optimal for precise measurements of Cl^−^ under physiological conditions. Furthermore, the relatively poor photostability of YFP derivatives hinders their application for dynamic and quantitative Cl^−^ measurements. Dynamic and accurate measurement of physiological concentrations of chloride would significantly affect our ability to study effects of chloride on cellular events.

**Methodology/Principal Findings:**

In this study, we developed a series of YFP derivatives to remove pH interference, increase photostability and enhance chloride sensitivity. The final product, EYFP-F46L/Q69K/H148Q/I152L/V163S/S175G/S205V/A206K (monomeric Cl-YFP), has a chloride K_d_ of 14 mM and pK_a_ of 5.9. The bleach time constant of 175 seconds is over 15-fold greater than wild-type EYFP. We have used the sensor fused to the transmembrane protein prestin (gerbil prestin, SLC26a5), and shown for the first time physiological (mM) chloride flux in HEK cells expressing this protein. This modified fluorescent protein will facilitate investigations of dynamics of chloride ions and their mediation of cell function.

**Conclusions:**

Modifications to YFP (EYFP-F46L/Q69K/H148Q/I152L/V163S/S175G/S205V/A206K (monomeric Cl-YFP) results in a photostable fluorescent protein that allows measurement of physiological changes in chloride concentration while remaining minimally affected by changes in pH.

## Introduction

Chloride is the major anion in cells, and plays various physiological roles. For example, chloride is a key determinant of intestinal fluid secretion and cell volume primarily through affecting osmotic gradients [1]–[3]. Chloride is also important in setting neuronal resting membrane potential through a number of chloride channels and inhibitory neurotransmitter receptors that have chloride conductance [4]. As a natural consequence of affecting these diverse phenomena many diseases result from disordered Cl^−^ regulation [5]. These diverse effects of chloride underscore the need to accurately and dynamically measure physiological intracellular chloride concentration.

While many proteins work to regulate intracellular Cl^−^, others are regulated by intra- and extracellular Cl^−^. For example, the unique membrane protein prestin in outer hair cells (OHCs), SLC26a5, functions as an ultrafast molecular motor, converting electrical to mechanical energy. This protein is thought to bring about cochlea amplification in mammals, which is responsible for the exquisite sensitivity of mammalian hearing. Intracellular chloride ions in the 0–10 mM range regulate the behavior of prestin, shifting its voltage responsiveness by −2 mV per mM of chloride (with gluconate as the counter anion). These *in vitro* findings have been supported by *in vivo* experiments demonstrating effects of chloride on cochlear amplification [6]. Hence, dynamic monitoring of intracellular Cl^−^ concentrations near prestin’s intracellular chloride binding site will aid in understanding chloride’s role in cochlear amplification. We previously have used the fluorescent dye MQAE to measure chloride flux in OHCs [7], but the technical problems with this approach are overwhelming. Consequently, we have worked to develop a genetically encoded chloride sensor that can be tagged on the intracellular C-terminus of prestin, sensing chloride fluctuation during prestin activity.

Over the last decade, engineered fluorescent proteins (FPs) were developed as genetically encoded indicators allowing non-invasive monitoring of intracellular ion fluctuations, such as Cl^−^ [8]–[11], pH [12], [13], and Ca^2+^ [14]–[16]. FPs are also quite useful for measuring membrane potential [17]–[19] and other cell biological phenomena. Most of them are GFP-based variants or FRET pair probes, like CFP-YFP.

EYFP, GFP-S65G/V68L/S72A/T203Y, has proven to respond rapidly and reversibly to concentration changes of halides, which enables YFPs to be genetically encoded Cl^−^ sensors in living cells. YFP-H148Q and its derivatives [8], including CFP-YFP-based Clomeleon [9], were introduced as genetically encoded chloride indicators, in which H148Q enhances the halide affinity due to better binding cavity access via a solvent channel, thereby favoring chromophore protonation following halide binding to reduce fluorescence [20]. The K_d_ of YFP at pH 7.5 is 777 mM, and 154 mM for YFP-H148Q, far removed from intracellular [Cl^−^] under physiological conditions. In the YFP-H148Q library, I152L and V163S exhibit higher chloride affinity with a K_d_ of 88 mM and 62 mM, respectively [10], [21], [22]. The triple mutant of YFP-H148Q/I152L/V163S was adopted in the CFP-YFP-based emission ratiometric Cl^−^ indicator (Cl-sensor) [11]. In Cl-sensor, the real sensor for chloride is the YFP mutant itself with K_d_ ∼30 mM, instead of requiring a peptide linker between CFP and YFP, or an extrinsic sensor such as calmodulin in Cameleon [23]. In YFPs, the halide-binding cavity near the chromophore has the ability to modulate the protonation state of the chromophore, and this is the basis of YFP’s chloride sensitivity. In the CFP-YFP based indicator, CFP is insensitive to halides, providing an *in situ* ratiometric calibration for YFP.

Compared to calcium FP-sensors, the YFP-based chloride sensor has less sensitivity (high mM scale instead of μM or nM), less photostability and is usually accompanied by significant, confounding pH effects near physiological pH. To date, YFP-based Cl^−^ indicators are limited in their application because of these three reasons. In this study, we developed a series of YFP mutants extending previous work. We introduced a positive charged residue, Q69K, into the halide-binding cavity to decrease pK_a_, and two folding mutations, F46L/S175G, to enhance folding. Furthermore, we uncovered a key mutation in the proton delivery pathway, S205V, which 1) increased the time constants of photobleaching 15-fold greater than wild-type EYFP, 2) lowered the pK_a_ away from physiological pH, and 3) enhanced the chloride sensitivity to 14 mM. These improvements provide for a superior chloride sensor. Finally, using this new enhanced chloride sensor, we demonstrate dynamic flux of chloride in the mM range into prestin expressing HEK cells.

## Results

### ClsM, a YFP Modification with Intermediate Chloride Sensitivity and pH Dependence

In designing a chloride sensitive YFP we evaluated and adopted a number of amino acid changes that had previously been shown to enhance fluorescence and stability. The maturation of YFP includes two phases, peptide chain folding and chromophore formation. F46L significantly accelerates chromophore oxidation, while the well-known folding mutations of F64L, M153T, V163A and S175G facilitate the folding process of the peptide chain [20], [24]. These mutations that affect folding and the rate limiting chromophore oxidation step also affect chloride sensitivity. F64L counteracts the conformational change in orientation caused by V68L in the YFP variant Venus, and also induces reduced halide sensitivity by preventing halide ion access to its binding site [24]. V68L is included in native EYFP, and we therefore opted to not include F64L in our new Cl^−^ sensor. Since V163A may also be involved in eliminating chloride sensitivity in Venus by presumed shortening of the side chain, we introduced V163S, with a longer side chain that enhances halide sensitivity [10] while simultaneously maintaining photostability. Similarly, S175G that breaks an existing hydrogen bond network facilitates folding *and* enhances fluorescence intensity of Venus [24], [25] and ECFP [25], and was incorporated in our constructs. M153T by virtue of its smaller side chain and increased flexibility [26] has also been shown to facilitate folding and increase fluorescence intensity. However, this mutation seemed to affect the expression of YFP in several of our constructs and was therefore not incorporated in our final constructs.

There are nine residues in the halide-binding cavity near the chromophore of YFP that have direct interaction or a distance of less than 5 Å from the binding halide anion, namely, Q69, R96, V150, I152, V163, F165, Q183, L201 and Y203 [20]. T203Y is the key mutational difference between YFP and GFP, and I152L/V163S already exist in the sequence of EYFP-H148Q/I152L/V163S from which we began our development effort. Q69 is fairly close to the chromophore anion inside the β-barrel of YFP. It was previously reported that Q69K could promote the anionic form of the chromophore to hinder its protonation, and therefore reduce the apparent pK_a_ to 6.1, with little effect on its other sensitivities [27]. But we found that the mutant Q69K when added to EYFP-H148Q/I152L/V163S folds poorly at 37°C, which was previously reported in EYFP-V68L/Q69K [27], possibly resulting from the extra length of the Lysine side chain and disturbing the hydrogen-bond network in the halide-binding cavity. Fortunately, this folding problem of Q69K can be compensated by the folding mutation of F46L. F46L can greatly accelerate the oxidation of the chromophore at 37°C, the rate-limiting step of maturation of chromophore, without changing the pK_a_ of EYFP [24]. The new construct, EYFP-F46L/Q69K/H148Q/152L/V163S, which we term **ClsM**, expressed very well in HEK-293 cells, showing bright fluorescence. The pK_a_ of **ClsM** was reduced to 6.5∼7.1 and was dependent on the chloride concentration (0.2 mM and 140 mM, respectively). The chloride sensitivity of **ClsM** remained at ∼30 mM (data not shown). Interestingly, the alternative replacement of Q69 with arginine could barely fold to exhibit fluorescence in transiently transfected HEK-293 cells, probably because of the large side chain of the arginine residue. In this study, all further mutants are made based on **ClsM**.

### Mutations to Reduce pH Sensitivity Resulted in Enhanced Photostability and Further Increased Chloride Sensitivity

Positive charges in the halide-binding cavity close to the YFP chromophore not only increase the affinity of halide binding, but also promote the anion state of the chromophore, which in turn can reduce the pK_a_ of the chromophore by dispersing negative charge over the conjugated structure of the chromophore. So our first strategy for improvement was introducing more positively charged residues into the halide-binding cavity.

We evaluated the addition of another positive charge besides Q69K into the halide-binding cavity of **ClsM** attempting to reduce confounding pH effects by single-site mutation of the individual residues V150, F165, Q183, L201 to lysine or arginine. However, all of the double-charge mutants (V150K/R, F165K/R, Q183K/R, L201K/R, each in combination with Q69K) could not fold to exhibit fluorescence, even in the presence of two additional folding mutations of M153T and S175G, as well as F46L. The double-charge mutants of Q69K/F165K and Q69K/L201K express somewhat better, but still showed lower than normal YFP fluorescence. We concluded that the double-charge residues in the halide-binding cavity can significantly affect the folding of YFP. We speculate that this could result from difficulty in burying an extra positive charge (in addition to R96 and Q69K) into the cavity proximal to the chromophore, due either to excess electrostatic forces or cavity size limitations.

**Figure 1:**
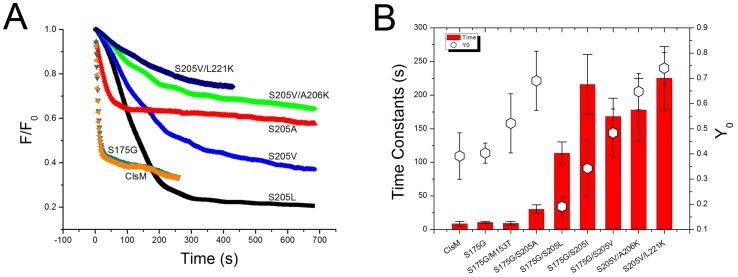
Photobleaching dynamics of YFP chloride sensor mutations. (**A**) Representative traces of some YFP variants photobleached at 430 nm. All variants are based on the EYFP-F46L/Q69K/H148Q/I152L/V163S (**ClsM**). Photobleaching data were fit by a single exponential decay function, 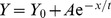, where Y_0_ is the constant offset representing the residual fluorescence after photobeaching (R^2^ ranged from 0.96–0.99). It is notable that the mutant ClsM-S175G/S205V/A206K (mClY, green curve) has a long time constant of 175 s under our photobleaching conditions, which is much longer than wild-type YFP and **ClsM** each at ∼10 s. (**B**) The red columns are the time constants of fluorescence decay during photobleaching, and Y_0_ is the constant offset of fluorescence exponential decay by photobleaching, representing the residual fluorescence when photobleaching reached a stable state. The folding mutations of S175G and M153T did not enhance the photostability of **ClsM**, while the mutations of S205 did increase the time constants of fluorescence decay.

The other strategy to lower the pK_a_ of the YFP-based chloride sensor is to set a barrier within the proton delivery pathway to the phenolate anion of the chromophore, which is protonated via a hydrogen bond network composed of the main chain of residue N146, the side chain of S205 and a bridging water molecule linked with a surface water molecule. The protons are delivered from the external solvent into the YFP β-barrel through this hydrogen bond network. If the proton delivery pathway is blocked or impeded, lower pH (higher [H^+^]) is needed to overcome the obstructed proton access, indicating that pK_a_ is reduced. We chose S205 as the essential target site to impede proton access. S205 was substituted with other neutral residues without hydroxyl groups, such as alanine, leucine, isoleucine, and valine. Interestingly, we found that in addition to its influence in altering pK_a_, S205 substitutions also enhanced photostability. Valine substitution of S205 has the best photostability. The percentage of residual fluorescence after photobleaching is the highest with this mutant S205V. Thus, the time constant of fluorescence photobleaching is 175 s compared to the **ClsM** bleaching time constant of ∼10 s (**Fig. 1**). Note that the folding mutations S175G or M153T does not enhance photostability.

Work from the Tsien laboratory has shown that the tendency for YFP dimerization can be greatly reduced or eliminated by mutating the hydrophobic amino acids in the dimerization interface to positively charged residues [28]. The order of effectiveness is A206K>L221K>F223R. The dissociation constant K_d_ of mYFP-A206K derived from the association constant (K_a_) is much higher than that of mYFP-L221K and mYFP-F223R [29]. Because prestin can oligomerize, we included the A206K mutation into the construct to make a monomeric version that will avoid aggregation when making fusion proteins with a targeting protein. Therefore, EYFP-F46L/Q69K/H148Q/I152L/V163S/S175G/S205V and its monomeric version with A206K, now termed **Cl-YFP** and **mCl-YFP (or mClY for short)**, respectively, were characterized in detail to determine their pK_a_ and chloride sensitivity.

**Figure 2:**
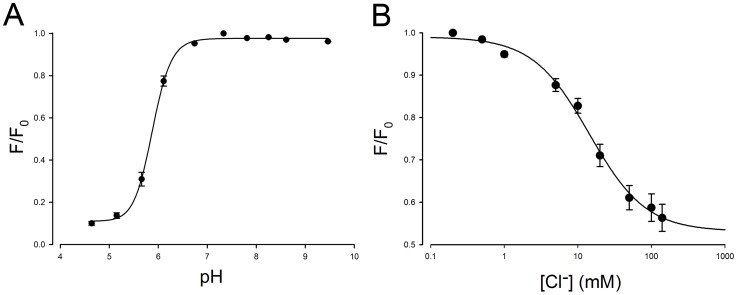
pH-sensitivity and chloride sensitivity of mCl-YFP measured by local perfusion of individual cells. (**A**) The pH-sensitivity of mCl-YFP fluorescence with a pK_a_ of 5.88 calibrated with a fixed 0.2 mM Cl^−^ solution containing 100 µM TBT and 50 µM Nigericin. F_0_ is the initial fluorescence at pH 7.20. For each data point, fluorescence data of more than 35 cells were averaged. Note in each case that the pK_a_ is far removed from physiological pH. (**B**) The chloride sensitivity of mCl-YFP with a K_d_ of 14.4 mM at a fixed 0.2 mM Cl^−^ solution using 100 µM TBT and 50 µM nigericin at pH 7.20. F_0_ is the initial fluorescence under 0.2 mM Cl^−^. For each data point, fluorescence data of more than 25 cells were averaged. The sensitivity is doubled compared to previous YFP-based Cl^−^ sensors, and within the physiological range of cellular Cl^−^ changes.

Cl-YFP and mCl-YFP are significantly less pH sensitive than ClsM, with a pK_a_ of ∼5.3 for Cl-YFP (data not shown) and a pK_a_ of ∼5.9 for mCl-YFP (**Fig. 2A**), both of which are far removed from physiological pH conditions. The pK_a_ of mCl-YFP is slightly higher than that of Cl-YFP, indicating better access for protons to reach the chromophore. This is likely due to a smaller steric barrier on the dimer interface. Although Cl-YFP and mCl-YFP have similar time constants for photobleaching, about 175 s, the percentage of residual fluorescence after bleaching was different; 50% and 65%, respectively (**Fig. 1**). Finally, mCl-YFP has a higher chloride sensitivity of 14.4 mM (**Fig. 2B**) compared to the previously reported best value of ∼30 mM for all YFP variants [11]. It should be noted that the larger reduction in fluorescence (F/F_0_) for low pH (**Fig. 2A**) versus high chloride (**Fig. 2B**) likely arises because protonation of the chromophore directly quenches the fluorescence of YFP, while chloride binding would change the proton affinity of chromophore, consequently reducing fluorescence intensity. Residual fluorescence that we measure likely depends on the oxidation equilibrium of fluorophor under the given spectrum and level of illumination such as optical power. It might be that if we changed these photobleaching conditions or solution pH that these asymptotic levels would differ. The point that we make is that under the same photobleaching conditions the relative response differs among mutations, with our mCl-YFP showing the best bleaching time constant, best pK_a_ and best K_d_ for chloride as well as the strong capability of removing YFP dimerization. The important point about bleaching is that it is minimized during the course of an experiment.

### The Outer Hair Cell Protein Prestin Shows Dynamic Chloride Movement that is Demonstrable with the Enhanced Chloride Sensor

**Figure 3:**
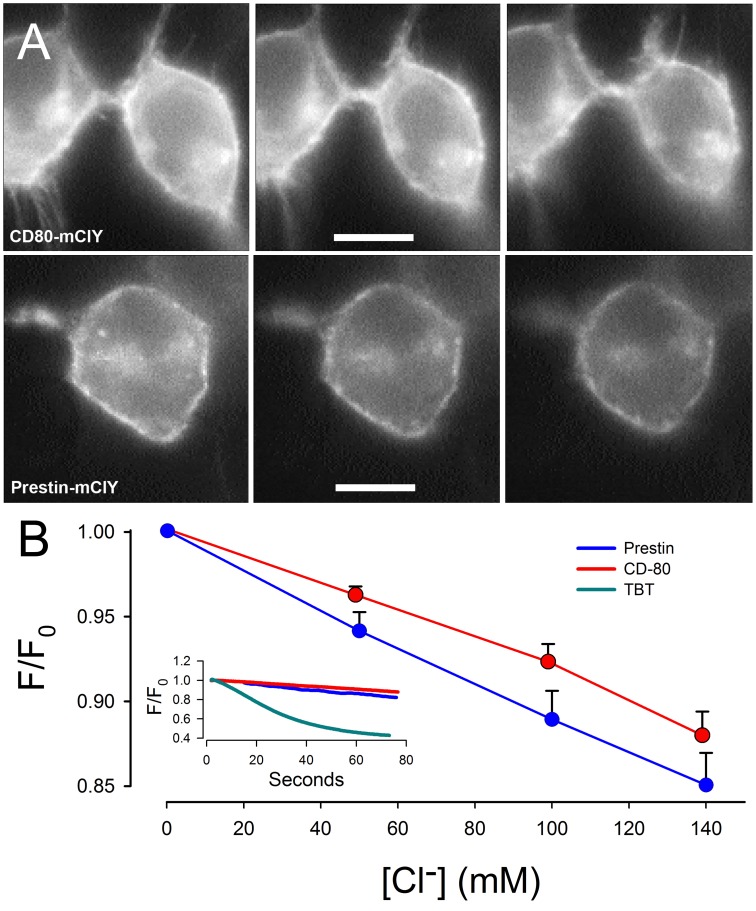
Prestin dependent Cl^−^ flux in HEK-293T cells. (**A**) Two panels show representative fluorescence change on HEK-293T cells transiently transfected with CD80-mClY (upper) and Prestin-mClY (lower), respectively, under local perfusion of high K^+^ solution with different Cl^−^ concentration, indicating a chloride flux into the cells. In the image panel, the perfused chloride concentration was <0.2 mM (left), 50 mM (middle), and 140 mM (right). All images were acquired at the same time. The bar in middle lane was 10 µm. These three lanes were in the same time scale. (**B**) The graph illustrates the effects on mean (+/−se, standard error) YFP fluorescence intensity upon local perfusion by High K^+^ (Na^+^-deficient) solution with different Cl^−^concentrations (19 cells expressing Prestin-mClY and 12 cells expressing CD80-mClY). The decrease in YFP fluorescence is more marked in cells expressing Prestin-mClY than CD80-mClY. All data have been compensated for photobleaching according to the photobleaching curve that was measured just prior to perfusion. Inset shows YFP fluorescence changes in two typical cells expressing Prestin-mClY and CD80-mClY, when perfused with 140 mM Cl^−^ from the bath solution containing 0.2 mM Cl^−^. This active but slower movement of Cl^−^ is contrasted with the rapid passive equilibration of intracellular Cl^−^ achieved by the addition of the ionophores TBT (100 µM) and nigericin (50 µM) while perfusing with 140 mM extracellular Cl^−^_._

To test our improved chloride sensor, mCl-YFP was fused to the C-terminus of the OHC motor protein, prestin, and transfected the construct into HEK-293T cells. After 24 hrs of incubation, chloride flux into the transfected cells was monitored during changes in extracellular chloride from 0.2 mM Cl^−^ to 140 mM Cl^−^ (**Fig. 3**). The standard bath solution contained 0.2 mM chloride. Upon perfusion with higher chloride solutions, the fluorescence dropped immediately demonstrating chloride influx. The most sensitive response was near the K_d_ of the sensor. Standard calibration of the sensor with 100 µM TBT and 50 µM nigericin provides a translation of fluorescence response to chloride concentrations. TBT and nigericin are ionophores that allow Cl^−^ and H^+^ ions to pass freely through the cell membrane.

In experiments using prestin-mClY, we noted a decrease in fluorescence upon exposure to increasing concentrations of extracellular chloride, compared to mCl-YFP fused to the control membrane protein CD80 (a B cell protein, the extracellular portion of which acts as a co-stimulatory molecule of T cells, and has no known chloride transport function). The relative reduction in fluorescence was proportional to the concentration of extracellular chloride applied to the cell. Although the differences in individual mean fluorescence did not reach statistical significance, the rates of change per extracellular Cl^−^ were significantly different (slope difference: 0.02 unit/100 mM Cl^−^; p<0.05), and indicates a more rapid transmembrane flux of Cl^−^ with prestin when extracellular Cl^−^ increases. The dilution of sub-membranous Cl^−^ into the cytosolic pool of Cl^−^ likely limits the accumulation of the anion near the plasmalemma. Importantly, prestin-mClY demonstrated unchanged prestin function evidenced in parameters of charge movement in the membrane (non-linear capacitance, data not shown). Moreover, there was tight correlation between estimates of intracellular chloride concentration determined by changes in voltage at peak capacitance (V_h_) [7], [30] and decrease in fluorescence intensity in prestin-mClY. Both methods of estimating Cl^−^ concentration are concordant with a rise in intracellular peri-membranous Cl^−^ concentration of <10 mM, when cells were perfused with 140 mM Cl^−^. Note that there was an increase in intracellular chloride concentrations with both prestin-mClY and CD80-mClY, although the reduction in fluorescence with prestin was more marked. We interpret these data to suggest that prestin enhances a basal Cl^−^ influx into the cell. The rise in intracellular juxta-membrane chloride concentration is due to a complex contribution from prestin activity (conductance or transport), native channels and transporters in the HEK cell, and diffusional dilution into the cytoplasm away from the plasma membrane. Resolving these issues will require additional work.

## Discussion

Here we report the development of a powerful fluorescent chloride sensor that displays 1) superior photostability, 2) reduced susceptibility to [H^+^] fluctuations near physiological pH, and 3) enhanced chloride sensitivity to permit assessment of low-level physiological changes in [Cl^−^]. For example, the K_d_ estimates of Prestin sensitivity to chloride range from 1–6 mM [7], [31], [32], and the fused Prestin/mCl-YFP sensor will be ideal for monitoring chloride levels in the OHC sub-plasmalemmal compartment where chloride concentrations have been speculated to fluctuate and affect prestin function [33]–[35]. Additionally, we demonstrate here dynamic changes in intracellular chloride concentration in response to changes in extracellular chloride that we attribute to prestin. It is unclear if this increase in intracellular chloride concentration is a result of transporter activity or a more channel-like conductance [36]–[38]. We are confident that the benefits of the probe will extend to other physiological preparations, as well.

### Protonation & Cl^−^ Binding

We used known characteristics of YFP and previous iterations of other chloride sensor homologues to engineer our new sensor. In previous published YFP variants, alterations in the Cl^–^ sensitivity is usually accompanied by alterations in confounding pH effects because halide binding promotes the protonation of the chromophore, and vice versa [8], [11], [20]. The relationship of pK_a_ and halide sensitivity of YFP variants exhibits features that imply positive cooperativity of protonation and halide-binding [8], [39]. In some variants of GFP, pH-sensitivity is exploited to measure compartmental pH [12], [40], but for FRET-based studies, the sensitivity of YFP to environmental pH is highly undesirable because it can interfere with the interpretation of the energy transfer efficiency or distance estimation between donor and acceptor. Indeed, YFP variants with low pH and halide sensitivity have been developed, namely Venus [24], [25] and Citrine [27]. Despite the apparent interdependence of pH and halide sensitivity, each can be separated.

In YFP, pK_a_ is mainly determined by two factors: the negative charge density on the phenolic oxygen of the chromophore and the local proton availability in the surrounding environment. The negative charge of chromophore anion mainly distributes on the phenolic oxygen or the carbonyl oxygen of imidazolinone as two major resonance structures. If the phenolate negative charge can delocalize over the conjugated skeleton to the carbonyl oxygen of imidazolinone, the phenolic oxygen would have less negative charge to attract protons for protonation, indicating that the pK_a_ would decrease. Our first strategy to decrease pK_a_ was to introduce as many positive charged residues as possible into the halide-binding cavity that is adjacent to the carbonyl oxygen of imidazolinone. Positive charges can help maintain the chromophore’s anionic state, and also likely provide a large fraction of anion-binding energy for increasing halide sensitivity. In our study, Q69K decreased the pK_a_ to 6.5–7.1 from 7.1–8.0 depending on the chloride concentration, and F46L resolved the folding problem caused by Q69K.

**Figure 4:**
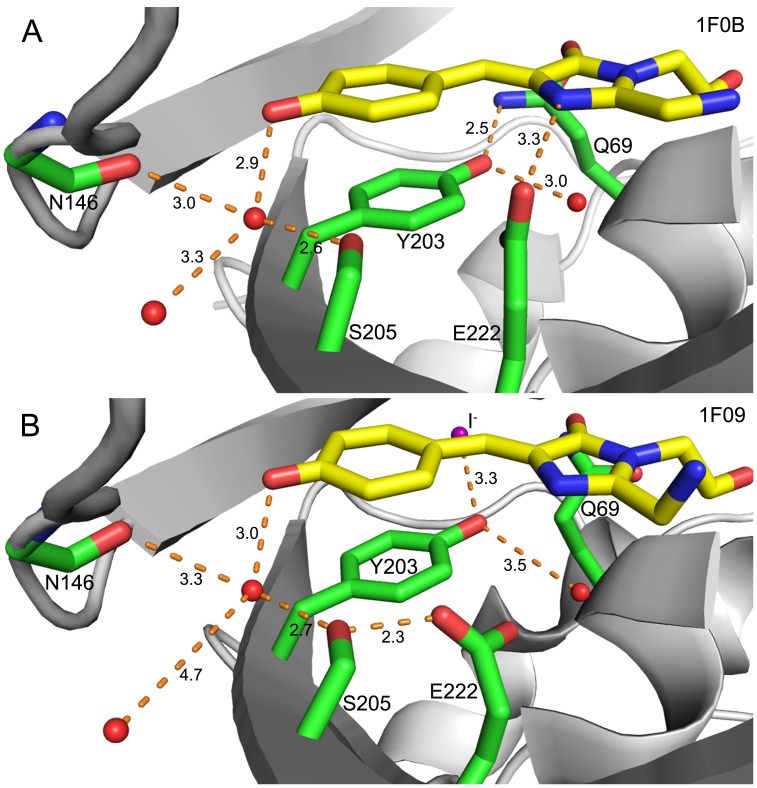
The H-bond network comprising the proton transfer pathway within the chromophore (in Yellow) of YFP-H148Q drawn with PyMol. Water molecules displayed as red balls. (**A**) YFP-H148Q without halide-binding (PDB: 1F0B). The phenolic oxygen of the chromophore is H-bonded to a water molecule that is also H-bonded to the side chain of S205 and the main chain of N146; additionally, there is a H-bond to a surface water molecule that is exposed to exterior solvent. E222 is H-bonded to the nitrogen on the imidazole ring, indicating that E222 is protonated, while the phenolic group of Y203 forms H-bond with Q69 and a nearby water molecule. Notably, there is no H-bond between S205 and E222 whose nearest distance is 3.9 Å. (**B**) iodide-bound YFP-H148Q (PDB: 1F09). The water molecule H-bonded to N146, S205 and the phenolic oxygen of the chromophore was separated away from the surface water molecule, whose distance increased to 4.7 Å. The deprotonated E222 forms H-bond to S205 that is in the H-bond chain with protonated chromophore, while the phenolic group of Y203 is near H-bonding distance with iodide, which may be a reason why YFPs are halide-sensitive since Y203 is the unique residue of YFP from GFP.

The local proton availability around the phenolic group of chromophore is proportional to the pH value of the external solution depending on proton accessibility. If the proton has unencumbered access to the phenolic anion of the chromophore, the pK_a_ will be higher. When proton access along the proton delivery pathway is encumbered, the pK_a_ value will decrease, indicating that a higher H^+^ concentration is needed to overcome the more difficult delivery. Therefore, the other strategy to decrease the pK_a_ was to identify mutations of some key residues that can block or raise the barrier within the proton delivery pathway to the phenol group of the chromophore. In the proton delivery pathway from the external solvent to the phenolic group of the chromophore, a water molecule adjacent to the chromophore phenolate forms H-bonds with the side chain of S205 and the main chain of N146, linking the chromophore phenolate with a surface water molecule within H-bond range (**Fig. 4**). Upon binding a halide ion, the distance between the bridging water molecule and the nearest surface water molecule increases to 4.7 Å, likely due to conformational rearrangement. S205 is the only residue that can be mutated to affect the bridging water molecule linked with a surface water molecule. Interestingly, S205 plays an important role for photostability, as it links E222 and the chromophore. In keeping with this possibility we also found that S205 mutations affect the photostability of the fluorophore, in addition to its effects on pK_a_.

### Photostability

Using selective screening assays and directed evolution strategies, highly photostable variants of mOrange and TagRFP were developed by Tsien’s group [41]. Nevertheless, the YFP photobleaching mechanism and details of the photo-reactive process remain poorly understood. We tried to endow our chloride sensor with higher photostability using a structure-guided strategy.

As noted above, we view S205 as being important for both proton delivery and photostability because of the hydrogen-bond chain network between the chromophore phenolate, S205 and E222 (**Fig. 4B**). Both GFP and YFP show decarboxylation of E222, evidenced as a loss of 44 Daltons (CO_2_), upon intense illumination, and in the case of YFP it is associated with photobleaching [42]–[44]. Continuous illumination irreversibly photobleaches YFP into a weakly fluorescent species, which absorbs at 390 nm and fluoresces at 460 nm, similar to its spectroscopic properties as free chromophore [42]; this behavior indicates that the photon-induced chemical destruction happens within the chromophore via excited states, while the protein is partially unfolding and aggregating [43], [44]. Though the detailed mechanism of how the E222 decarboxylation of YFP induces chromophore destruction remains to be determined, it could involve the hydrogen bond network between the chromophore and E222. In YFP-H148Q, E222 could be either H-bonded to S205 or to the nitrogen on the imidazole ring of the chromophore, but not to both at the same time (**Fig. 4**).

We found that the mutation S205V increased YFP photostability (bleach tau = ∼175 s) more than 15 fold over wild-type YFP or **ClsM**. In fact, not only is its photostability enhanced, but the possible H-bond network rearrangement afforded by the mutation of S205 reduces pK_a_, and improves chloride sensitivity, indicating that a new equilibrium was reached between the chromophore protonation and halide-binding. S205A, S205L and S205I similarly cannot support proton transfer in the absence of a hydrogen bond donor or acceptor at position 205. S205A, which is less bulky than S205V, provides a bleach time constant of ∼30 s. S205L and S205I, which have larger side chains than S205V, show bleach time constants of ∼110 s and ∼220 s, respectively. This may indicate that side chain size or rotational freedom of residue 205 is important for photostability.

Interestingly, it was reported that wtGFP-S205V and wtGFP-S205A slow down the travel time through the excited-state proton transfer pathway (ESPT) from several tens of picoseconds to a few nanoseconds by rearranging E222 and Thr203 to form an alternative ESPT pathway without S205 [45], [46]. It is unlikely that this would occur in YFP because it lacks the corresponding neutral form of the chromophore that wtGFP possesses, and the orientation of Y203 in YFP cannot permit its phenolic group to interact in an alternative H-bonding network for proton transfer. Furthermore, any potential effects of these mutations on GFP photobleaching were not reported.

In summary, we have developed an YFP-based chloride sensor that has enhanced chloride sensitivity and photostability, while possessing reduced confounding pH effects. We have used it to measure sub-membranous chloride flux in HEK cells when fused to the transmembrane protein prestin, and show that it is capable of monitoring changes in intracellular chloride at levels expected to have physiological impact. We also note that the stability of our YFP mutants could be useful in studies where photobleaching plays a key role, for example in single molecule [47] and superresolution microscopy [48] methodologies.

## Methods

### 1. Gene Construct and Mutations

Mutagenesis was performed using the Quick Change method adapted from Stratagene QC protocol. Mutations were verified by sequencing the entire gene. The vector is EYFP-N1. The sequences of EYFP-H148Q/152L/V163S and ClsM were synthesized by GeneWiz (USA).

### 2. Cell Culture & Excitation Ratiometric Imaging System

HEK-293T cells were cultured on glass coverslips (No. 1, 0.13–0.16 mm thick, 15 mm round, Warner Instr., USA), which were mounted on a quick change chamber and platform (RC-42LP and QE-1, Warner Instr., USA). Transient transfection with the plasmid of interest was done using lipofectamine-LTX with Plus Reagent (Invitrogen, Life technologies). Fluorescence images were acquired using an oil-immersion objective (N.A. = 1.30, Plan Fluor 100× Objective, Nikon, Japan) with a Nikon Eclipse Ti equipped with a 200W metal-halide lamp (Lumen200, Prior Scientific, USA) as the fluorescence illumination source. Shutter and filter wheel (Lambda10–3 optical filter changer with smart shutter, Sutter Instr., USA) were connected between the microscope and the illumination source, in which Semrock ET430/24x-32 was used as the excitation filter for CFP, Semrock ET500/20x-32 as the excitation filter for YFP and Chroma HQ520LP as the emission filter. A 14-bit back-illuminated EMCCD camera system (128×128 pixels, 24 µm array, Andor iXon^EM^+ DU-860E, USA) was used to record the fluorescence images under CFP or YFP excitation. All peripheral hardware control, image acquisition and image processing were achieved and/or synchronized on a PC computer via a 16-bit/1-MHz USB Data Acquisition System (Personal Daq/3000 Series, IOtech, USA) by using customized software (jClamp & FastLook, SciSoft, USA; www.SciSoftCo.com). The average fluorescence intensity of regions of interest (ROI) was measured, and the background fluorescence was subtracted using ImageJ. The excitation ratios (*F*_500_/*F*_430_) of fluorescence intensity were then determined. The emission spectrum of mCl-YFP has the same shape as wt-YFP, with the peak around 527 nm. This was determined at an excitation of 485 nm using a microplate reader (TECAN infiniti M1000 Pro). The determinations of photobleaching, pK_a_ and chloride sensitivity were made using HEK-293 cells expressing YFP mutants directly in the cytosol, and not with membrane bound fusion proteins, which avoids the possibility of confounding results caused by limited expression or dim fluorescence. Data were analyzed with Matlab, Origin 8.0 and SigmaPlot 10.0.

### 3. Photobleaching

Photobleaching data and fluorescence images were achieved with our ratiometric imaging system controlled by jClamp & FastLook. Photobleaching efficiency at a wavelength of 500 nm is lower than at 430 nm, although the absorption at 430 nm is much lower than 500 nm. Because of this enhanced bleaching capability and to optimize our identification of photostable products, we bleached at the CFP excitation wavelength of 430 nm (approximately 17 µW). At the beginning of every episode, the excitation filter was changed to the YFP filter (500 nm) by the filter wheel and an image was captured by the camera and then the excitation filter was changed back to CFP filter (430 nm) for bleaching until the next acquisition. Filter change and acquisition took about 100 ms. The protocol includes 400 episodes and 200 ms interval time between each episode. CFP excitation remained on during intervals. Stable optical power at the utilized wavelengths was confirmed using an analog optical power meter (ThorLabs PM30–130, w/S130A Slim Sensor). Photobleaching of mCl-YFP did not shift its emission peak.

### 4. pK_a_ Measurement

The pK_a_ of the YFP mutants were directly measured from the fluorescence change of the transfected HEK-293T cells under local perfusion (Y-tube) with low [Cl^−^] solutions (0.2 mM Cl^−^) at different pH values containing 50 µM nigericin and 100 µM TBT, which eliminates pH and Cl^−^ gradients across the cell membrane, respectively. A Hill function (SigmaPlot, unconstrained, 4 parameters, 
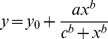
) was fitted to the data points to calculate the apparent pK_a_.

### 5. Chloride Sensitivity Calibration

The chloride sensitivity of YFP mutants was measured from the fluorescence change of the transfected HEK-293T cells under local perfusion (Y-tube) with near neutral solutions (pH 7.20) of different [Cl^−^] containing 50 µM nigericin and 100 µM TBT, according to the standard nigericin-tributyltin equilibrating protocol. A Hill function (SigmaPlot, unconstrained, 4 parameter) was fitted to the data points to calculate the apparent K_d_. Local perfusion were performed with high K^+^ solution (Na^+^-deficient) to minimize any pH effect on the chloride sensor by native membrane Na^+^/H^+^ exchanger.

### 6. Chloride Flux Promoted by Local Perfusion

The chloride flux into HEK-293 cells expressing prestin-mClY or CD80-mClY was measured by fluorescence change during local perfusion using high K^+^ solutions containing different chloride concentrations at pH 7.20. Photobleaching compensation was corrected based on the photobleaching time constant that was measured prior to Cl^−^ perfusions. ImageJ was used to define a region of interest around membrane sections that were free from movement artifact in which fluorescence intensity changed with perfusion with different Cl^−^ concentrations. Perfusion data were analyzed using mixed model repeated measurements with a group-specified compound symmetry structure within SAS software. The repeated-measures design was used for the experiment of comparing the Cl^−^ influx pattern between prestin and CD80 constructs. Since each cell was perfused with a series concentration of extracellular Cl^−^, fluorescence measured within each cell was correlated. A repeated measures analysis using the procedure of Proc Mixed in SAS software (Cary, NC) was performed to model the change of fluorescence per extracellular Cl^−^. The dependency between repeated measures for same cell was incorporated into analysis by using a group specified compound symmetry covariance structure, which assumes common variance and covariance within group and accounts for the heterogeneous structure between groups. The interaction between group and concentration of Cl^−^ was included in the model to examine the difference of rate of change in fluorescence between groups [49], [50].

### 7. Data Availability Statement

All data are supplied in the manuscript, including information on point mutations. Further requests can be made to the corresponding author.
